# Neurological Pain, Psychological Symptoms, and Diagnostic Struggles among Patients with Tick-Borne Diseases

**DOI:** 10.3390/healthcare10071178

**Published:** 2022-06-23

**Authors:** Sarah P. Maxwell, Chris Brooks, Connie L. McNeely, Kevin C. Thomas

**Affiliations:** 1 School of Economic, Political & Policy Sciences, University of Texas at Dallas, Richardson, TX 75080, USA; 2 Laboratory for Human Neurobiology, Boston University School of Medicine, Boston, MA 02118, USA; crbrooks@bu.edu (C.B.); kipthoma@bu.edu (K.C.T.); 3 Center for Science, Technology, and Innovation Policy, George Mason University, Fairfax, VA 22030, USA; cmcneely@gmu.edu

**Keywords:** tick-borne disease, pain, neurological symptoms, psychiatric symptoms, Lyme disease, diagnostic burden

## Abstract

Public health reports contain limited information regarding the psychological and neurological symptoms of tick-borne diseases (TBDs). Employing a mixed-method approach, this analysis triangulates three sources of symptomology and provides a comparison of official public health information, case reports, medical literature, and the self-reported symptoms of patients with Lyme disease and other TBDs. Out of the fifteen neuropsychiatric symptoms reported in the medical literature for common TBDs, headaches and fatigue and/or malaise are the only two symptoms fully recognized by public health officials. Of TBDs, Lyme disease is the least recognized by public health officials for presenting with neuropsychiatric symptoms; only headaches and fatigue are recognized as overlapping symptoms of Lyme disease. Comparisons from a patient symptoms survey indicate that self-reports of TBDs and the associated symptoms align with medical and case reports. Anxiety, depression, panic attacks, hallucinations, delusions, and pain—ranging from headaches to neck stiffness and arthritis—are common among patients who report a TBD diagnosis. Given the multitude of non-specific patient symptoms, and the number and range of neuropsychiatric presentations that do not align with public health guidance, this study indicates the need for a revised approach to TBD diagnosis and for improved communication from official public health sources regarding the wide range of associated symptoms.

## 1. Introduction

Patients who are diagnosed with Lyme disease (LD) or other tick-borne diseases often develop chronic or long-term symptoms, leading to debates over the use of terms such as “chronic Lyme” and “post-treatment Lyme disease syndrome” (PTLDS). According to the Centers for Disease Control and Prevention (CDC) in the United States, when Lyme disease and other tick-borne diseases are not treated early, PTLDS may occur from autoimmune phenomena, with or without persistent infection, resulting in pain, brain fog, and fatigue more than six months after antibiotic treatment [1]. However, it is also known that 10–20% of patients who are treated in a timely fashion will also develop PTLDS [2]. Numerous studies report multisystem and severe symptoms that substantially impede a patient’s quality of life [3,4,5]. The psychological distress of chronic illness in patients with Lyme disease in particular can be bi-directional, resulting in further diagnostic burdens for patients and medical providers [3,4]. In other words, patients who are ill, as with any chronic or serious condition, could become depressed from the burden of the illness alone, in addition to a reaction from the pathogen itself.

- This research explores the medical literature related to tick-borne disease (TBD) symptomology, in comparison to official public health reports and to survey respondents who reported a diagnosis of one or more TBD. These comparisons contribute to the study of TBDs by analyzing the symptoms noted by patients and medical reports but are not recognized by public health officials.
- This study presents patient self-reports regarding their diagnosis and quality of life, specifically regarding psychological distress and concomitant neurological symptoms.

### 1.1. Burden of Diagnosis: Controversial Pathogenic Insults

Tick-borne diseases (TBDs) in the United States account for 77 percent of all vector-borne diseases, as reported by the National Notifiable Diseases System (NNDS), 2004–2016 [6]. Tick-borne diseases represent a varied set of infections that can be caused by bacteria, viruses, and protozoa [7]. Tick-borne infections are “under-appreciated causes of central nervous system (CNS) infection”, as a result of under-diagnosing and under-reporting [7]. LD or Lyme Borreliosis (LB), a tick-borne pathogen causing multisystem symptoms [8,9] in humans, is estimated to account for 476,000 cases per year [10], suggesting that the actual number of TBD cases may be substantially higher, indeed, more than ten times higher than stated in the official reports [7]. Other TBDs have expanded geographically, beyond regions considered endemic, resulting in increased disease cases, ranging from spotted fever rickettsiosis to anaplasmosis and babesiosis [6]. Global public health, as a field, is recognizing the burden of TBDs, which are increasing as a result of both abiotic and biotic factors [11]. Clinical presentations of tick-borne diseases vary, but not uncommonly, TBDs clinical presentations involve CNS manifestations, which include palsy, confusion, meningoencephalitis, encephalitis, meningitis, and encephalopathy [7].

In addition to hard-bodied ticks that spread LD and other TBDs, recent attention is also being paid to tick-borne relapsing fevers (TBRFs), known to be caused by the soft-bodied or Argasidae tick vectors. However, TBRFs can also be transmitted by hard ticks or Ixodidae vectors [12]. Relapsing fever borreliae, such as LD, are spirochaetes. Spirochetal illnesses, such as syphilis and LD have been referred to as “great imitators,” given their symptom similarities with other diseases. For example, often multisystem and nonspecific, syphilis and LD can both result in numerous neuropsychiatric symptoms. In fact, syphilis patients accounted for twenty-five percent of the psychiatric beds at the beginning of the 20th century [13].

### 1.2. Testing for TBDs, EM Rash, and Diagnostic Struggles

Testing for Lyme disease is particularly controversial given false negative and false positive occurrences. False positives may occur with other spirochetal illnesses, such as syphilis and periodontal disease, as well as infection with the Epstein–Barr virus (EBV) [13,14,15]. Serological testing faces numerous challenges, including the time it takes for a person to generate antibodies, a lack of sensitivity following antibiotic treatment, and the test’s inability to distinguish an active infection from mere past exposure [6,13].

The CDC guidelines are based on serology, but it is common for a patient not to produce antibodies. The resulting test will, therefore, produce a false negative in cases where the infection is acute and the test is performed too soon. False negatives may also occur when a patient is either immune compromised or had received treatment early after contracting a TBD [16,17]. For those with late-stage LD or other TBDs, a false negative is notably relevant to neuropsychiatric sequelae.

Additionally, tick saliva injected into a human host not only carries molecules to reduce histamine, but also carries pain-reducing molecules, meaning that the host may not realize that they have been bitten. In addition, it also introduces molecules that can elude white blood cells, effectively undermining the host’s immune response [18]. A lack of antibody production by some human hosts is problematic in current testing [19], leading some patients to seek out specialty labs, which many physicians and the CDC consider to be unconventional testing. Serologic testing is often negative in early-stage Lyme disease as well, which suggests that treatment should be administered if an EM rash is present [7].

The diagnostic struggles for patients with a TBD, or in whom a TBD is suspected, can cause a significant economic burden, present difficulties for patients in search of a diagnosis, and can lead to long-term impacts on patients’ quality of life. At least 3.4 million Lyme disease tests are performed by specialty laboratories in the United States each year [20,21]. Physicians often fail to test for TBDs, other than Lyme disease, as it is the most recognized. One study found that 75.6% of family practitioners underestimated the incidence rates of erythema migrans (EM) rashes, and approximately one-fourth were aware that the presence of an EM rash, alone, was diagnostic for LD [22]. In endemic areas, EM rashes alone often are used in clinical diagnoses [23]. These findings support a more recent study on long-term patients, which concludes as follows: “Although physical exams and clinical laboratory tests showed few objective abnormalities, standardized symptom questionnaires revealed that patients with PTLDS are highly and clinically significantly symptomatic, with poor health-related quality of life” [5].

Adding to the diagnostic burden, EM rashes are not always present, nor do all patients remember receiving a tick bite [6]. Additionally, the classic “bull’s-eye” EM rash does not appear in many cases [24] and, even when an EM rash is present, clinicians may not recognize it as a symptom of LD. In children, clinicians missed 12% of LD cases that presented with an EM rash; furthermore, they also believed 31% to have LD when they actually did not have the disease [25]. Misunderstandings about the necessity of a bull’s-eye rash, in addition to intermittent symptoms, can also delay diagnosis [26].

Many TBDs other than Lyme disease do not present with the bull’s-eye rash [27]. TBD diagnosis is challenging, especially in areas perceived to be non-endemic where medical practitioners may not be alert to related symptoms [28]. Patients who are left untreated may develop severe neuropsychiatric symptoms and decreased quality of life. In comparison to patients with other chronic illnesses, LD patients present particularly poor quality of life outcomes [3,29,30]. The International Lyme and Associated Diseases Society supports the use of clinical diagnoses, considering the diagnostic challenges [31]. However, case studies of patients being misdiagnosed with LD are not uncommon, such as reports of patients with neoplasms, whose medical treatment was delayed as a result of an LD diagnosis [23].

Late-disseminated LD and other TBDs can cause neurologic, psychiatric, and influenza-like symptoms, accompanied by numerous other multisystem and often debilitating symptoms, resulting in an extremely poor quality of life [3]. Misdiagnoses are more common in urgent care and emergency room settings, and occur as a result of symptom misattribution, symptoms that are intermittent, and of some physicians’ belief that a bull’s-eye rash must be present [26]. The controversial and ongoing debate regarding the clinical diagnosis of Lyme disease begets further diagnostic struggles, as clinicians may fail to test for tick-borne related co-infections, such as rickettsiae or babesiosis, as well as other diseases, such as Chlamydia pneumoniae, with similar clinical presentations [32]. In a large US study, some LD patients waited 10 years for the correct diagnosis and many reported having to travel more than 50 miles for treatment [29].

## 2. Materials and Methods

### 2.1. Tickborne Diseases of the United States: A Reference Manual for Health Care Providers

The complexity of diagnosis originates from patients presenting with non-specific and multisystem symptoms, with potential misattribution of symptoms by practitioners, regarding psychiatric and associated neurological problems. Using a mixed- methods approach, the combination of methodological approaches included a systematic review of the literature on psychiatric and neurological symptoms, in addition to a comparison of the case reports and the medical literature, with the Centers for Disease Control and Prevention’s manual: *Tickborne Diseases of the United States: A Reference Manual for Health Care Providers* [27]. The articles were pulled from English language journals found in PubMed. Only those which presented case reports or research related to TBD in the United States were included; case reports from Europe and other countries were excluded as tick-borne pathogens may cause different symptoms.

### 2.2. CDC Guidance, Medical Literature, and Patient Self-Reports

These findings are further detailed through comprehensive comparison tables that collect all CDC-reported symptoms for all tick-borne diseases, as documented and shared with health providers. This listing entails a wide range of reported symptoms from a variety of commonly acquired tick-borne diseases, as reported by the CDC. A second table includes all CDC-reported psychological and neurological symptoms, comparing the CDC guidelines for health care providers to the literature found via PubMed. To date, less information is available on the psychiatric and neurological symptoms that extend beyond the CDC-recognized symptoms of “headache” or “altered mental state”. Through a comprehensive search via PubMed, the symptoms are identified and summarized to assess differing and dispersed knowledge regarding possible presentations of Lyme disease and other tick-borne diseases.

Through a mixed-methods approach, 148 patients who self-reported having Lyme disease or another TBD were surveyed using a convenience sample. The patient symptom survey (PSS) was created using Qualtrics, an online survey platform. An anonymous survey link was provided via Facebook and was distributed widely via numerous “shares” among individuals and tick-borne disease organizations. The survey was designed to reach potential respondents who had a diagnosis of LD or another TBD, as they are most likely to frequent health and LD-related organizations online. The survey reached respondents who voluntarily completed questions regarding their self-reported diagnosis and resulting symptoms. Frequencies of psychiatric and neurological symptoms by disease type have been reported here to further assess differing public health and case reports and to inform public health practitioners about the possibility of unusual presentations of Lyme disease and other TBDs. Although there are limitations to using patient self-reports, with limited data regarding the incidence of certain symptoms, survey reports are becoming more commonplace [3,28]. Including patient responses in the literature review was intended to serve as an exploratory approach to determine self-reported symptoms. Given the online nature of the survey, respondents were not asked to verify their diagnosis through laboratory results.

The overall framework was holistic and exploratory to look across outcomes of symptomology using a triangulation approach in comparing differing methods, including the official guidance, case reports, and medical literature, and patient self-reports from a national sample. The patient symptom survey was approved by the Ethics Committee under the Declaration of Helsinki Institutional Review Board Guidelines. Informed consent was obtained from all subjects involved in the study. The authors declare no conflict of interest.

## 3. Results

### 3.1. Neurological Symptoms and Sequalae of LD and TBDs

“Lyme neuroborreliosis (LNB) is the neurological manifestation of the systemic infection caused by the spirochete Borrelia burgdorferi (BB) sensu lato” [33]. Lyme borreliosis encompasses all areas of the nervous system, with as many as 20% developing neurological symptoms [34,35]. Below, a review of the key symptoms is presented. The last row for each symptom provides information from the Centers for Disease Control to provide a comparative reference to the scientific literature. Specifically, the CDCs *Tick-borne Diseases of the United States: A Reference Manual for Healthcare Providers* is referenced for each symptom [27]. The importance of symptom recognition in TBDs cannot be overstated, as they are essential for proper testing and diagnosis. When discrepancies between the scholarly literature and official sources exist, the diagnostic burden for those with TBDs increases (Table 1, Table 2, Table 3, Table 4, Table 5, Table 6, Table 7, Table 8, Table 9, Table 10 and Table 11).

### 3.2. TBD and Psychiatric Symptoms and Sequelae

Psychiatric manifestations are reported among patients with LD and co-infections or other TBDs (Maxwell, 2020 [3]). Hájek et al. (2002) [111], for example found the following: “Higher prevalence of antibodies to Borrelia burgdorferi in psychiatric patients than in healthy subjects”. Bransfield (2018) [67] noted the following: “There is increasing evidence and recognition that Lyme borreliosis, and other associated tick-borne diseases (LB/TBD) cause mental symptoms” (p. 1). Fallon et al. (2021) provided insights on patients diagnosed with LB in hospital settings in Denmark, noting the increased risk of suicide and suicide attempts, and other mental disorders, in a Danish cohort study [112]. However, the literature is generally scant in disaggregating specific psychiatric manifestations. Searches for “depression” and “tick-borne”, for example result in canine studies or those related to Tick-borne encephalitis, a disease primarily found in Europe and Asia. Overall, the psychiatric symptoms related to TBDs have been largely ignored. The literature review below includes psychiatric manifestations beyond depression and anxiety and extends to hallucinations and delusions. Fatigue and malaise are included in this section, as they are common symptoms of TBDs, generally, but are also linked bi-directionally to patients with Lyme disease and other TBDs (Table 12, Table 13, Table 14 and Table 15).

The clinical outcomes for patients with tick-borne diseases are largely unrecognized, even though Lyme disease is the most common vector-borne disease in the United States [62]. Lyme disease is a catch-all term used by many LD organizations to characterize a host of tick-borne illnesses. As a result, patients may request Lyme disease testing, which may not be conclusive or may result in negative laboratory findings if the patient is infected with a different TBD. “Patients with LD are more likely to report nonspecific long-term sequelae, especially those experiencing persistent symptoms posttreatment” [62]. Given the varying and non-specific clinical presentations and the long-term outcomes of tick-borne diseases, clinicians referring to the CDC guidelines may miss additional symptoms, especially those with neuropsychiatric involvement. Below, the full range of symptoms for every TBD reported by the CDC is summarized in Table 16.

Using the systematic literature review and the CDC summary table, Table 16 offers a symptom comparison for tick-borne diseases that are more common in the US and within the medical literature. Specifically, the focus includes the following: LD, BVD, babesiosis, ehrlichiosis, tularemia, anaplasmosis, RMSF, and PVD. Table 17 summarizes the neuropsychic symptoms used in the present study’s survey of LD patients, who reported tick bites and subsequent TBDs.

The comparison summary in Table 17 indicates that out of the fifteen neurological symptoms reported in the medical literature, headaches and fatigue, and/or malaise are the only two symptoms that the CDC fully recognizes in accordance with the scholarly studies and case reports. Of those fifteen more common neuropsychiatric symptoms, Lyme disease is the least recognized TBD by the CDC for presenting neuropsychiatric symptoms, and the CDC does not acknowledge eleven symptoms found in the literature. Only headaches, fatigue, and pain were reported by the CDC as over-lapping symptoms of LD. The neurological symptoms that were least recognized by the CDC but were reported in scientific studies included the following: difficulty swallowing (dysphagia), speech impairment (dysarthria), low blood pressure (hypotension), fainting, vertigo and dizziness, and seizures.

### 3.3. Patient Symptom Survey Results

A total of 239 respondents completed the online survey before the predetermined cut-off date of 31 March 2021. Only respondents who reported at least one tick-bite encounter in a United States county after 1 January 2000 were included in the final analytical cohort. The final analytical cohort consisted of 148 respondents (61.92% of all respondents), spread over 144 US counties. Of these 148, 81 (54.73%) reported a diagnosis of anaplasmosis, babesiosis, ehrlichiosis, Lyme disease, and/or Rocky Mountain spotted fever. The analytical cohort included a wide range of ages and an approximately equal number of males and females, although these varied within each diagnostic cohort. Notably, ~83% of all respondents who reported a TBD diagnosis were female, despite making up only ~46% of the analytical cohort (Table 18).

**Figure 1:**
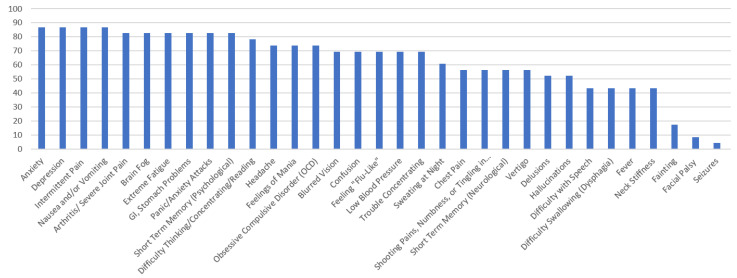
Percentage of Respondents with Ehrlichiosis Reporting Specific Symptoms.

**Figure 2:**
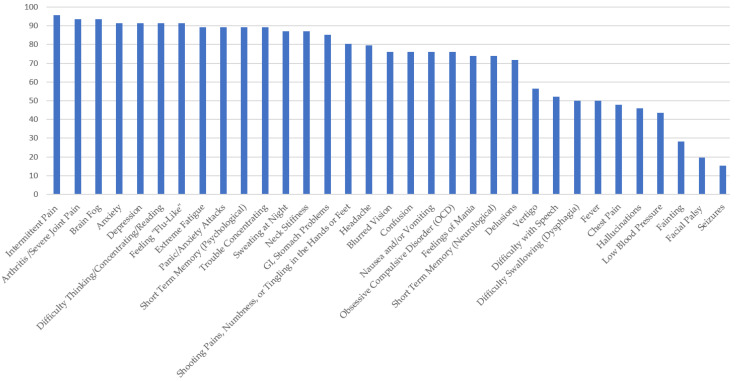
Percentage of Respondents with Babesiosis Reporting Specific Symptoms.

**Figure 3:**
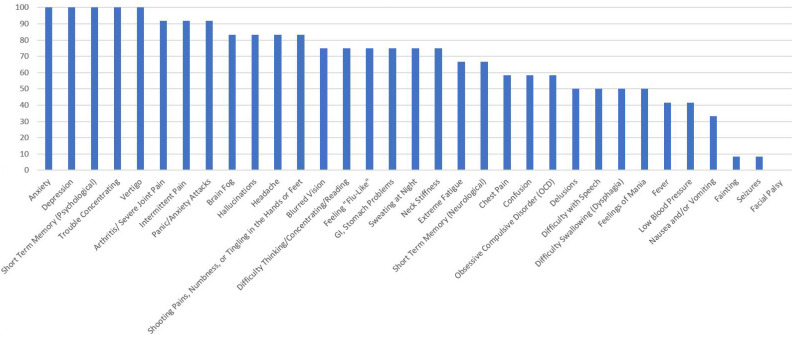
Percentage of Respondents with Anaplasmosis Reporting Specific Symptoms.

**Figure 4:**
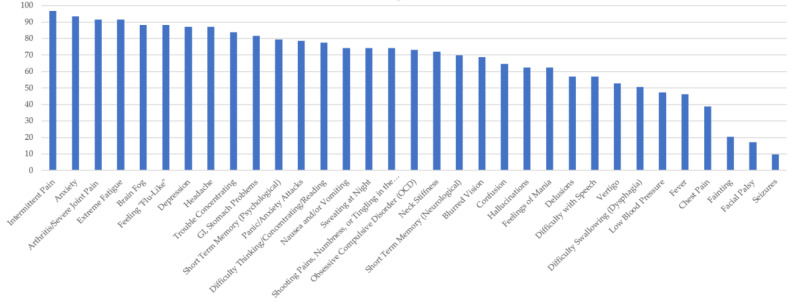
Percentage of Respondents with Lyme disease Reporting Specific Symptoms.

**Figure 5:**
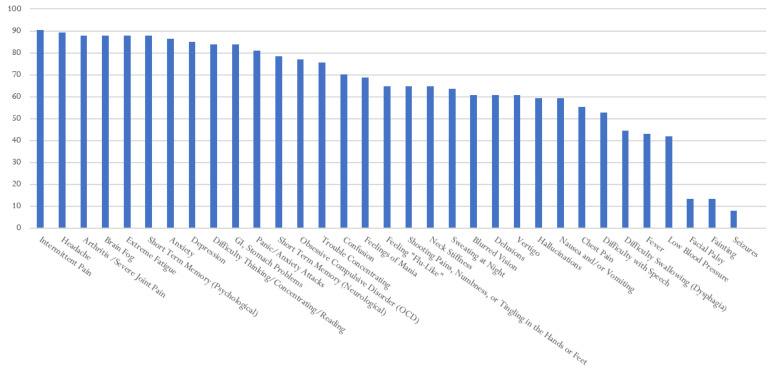
Percentage of Respondents with RMSF Reporting Specific Symptoms.

Figure 1, Figure 2, Figure 3, Figure 4 and Figure 5 provide the results from the patient symptom survey, comprising 148 respondents from across the United States who reported a diagnosis of a tick-borne disease. As is evidenced from Figure 1, Figure 2, Figure 3, Figure 4 and Figure 5, there is a wide diversity of noted tick-borne disease outcomes, which impact the physiological and psychological reported symptomology. Among all of the respondents, symptoms were evident across all categories of reported conditions, with the least-reported in this sample being facial palsy, seizures, fainting, and fever. For each TBD, the rates of the symptoms reported in this survey demonstrated that the most often reported diseases were Lyme disease, followed by Rocky Mountain spotted fever, babesiosis, ehrlichiosis, and anaplasmosis, with specific occurrences of underlying symptomology, respective to each TBD. The charts, below, provide the percentages of respondents who indicated that they experience each symptom, organized by disease diagnosis.

The survey respondents who reported a diagnosis of ehrlichiosis point to very high levels of neuropsychiatric symptoms. The most prominent (over 80% of the reports) were as follows: arthritis, with severe joint pain and swelling (particularly the knees and other large joints); brain fog, headache, and short-term memory. The following symptoms accounted for over 90% of the reports: anxiety; depression; GI/stomach problems; intermittent pain in tendons, muscles, joints, and bones; nausea and/or vomiting; and panic attacks. The most common symptoms involved pain, GI, and psychological symptoms, specifically those related to anxiety and depression. Only one respondent reported Bell’s palsy or seizures, and only two reported fainting.

In Figure 2: Survey respondents who reported a diagnosis of babesiosis experienced symptoms similar to ehrlichiosis, but at higher frequencies. More than 90 percent of those surveyed reported the following: arthritis, with severe joint pain and swelling; intermittent pain; anxiety; depression; brain fog; headaches; feeling “flu-like”; trouble concentrating; and extreme fatigue. Over 80% reported shooting pains, numbness or tingling in the hands or feet, hallucinations, obsessive compulsive disorder (OCD), nausea and/or vomiting, sweating at night, difficulty thinking/concentrating/reading, and GI (stomach) problems. Facial/Bell’s palsy and fainting were the least reported symptoms among those who self-reported a diagnosis of babesiosis.

Figure 3 shows the percentage of PSS respondents with anaplasmosis, who reported neurological, psychiatric, and pain-related symptoms. One hundred percent of those with a self-reported diagnosis of anaplasmosis reported anxiety, depression, short-term memory and concentration difficulties, and panic attacks. Over 85% also reported the following: hallucinations, obsessive compulsive disorder (OCD), arthritis, brain fog, headaches, extreme fatigue, and intermittent pain.

Figure 4 shows the survey respondents who reported a diagnosis of Lyme disease. LD respondents experienced similar symptoms to all TBDs, at frequencies of over 90 percent for intermittent pain, anxiety, and extreme fatigue. Arthritis and joint swelling, feeling flu-like, brain fog, depression, and headaches were also reported frequently (~87%). Patients with LD in this study reported numerous neuropsychiatric issues. For Lyme disease respondents, only a few symptoms were reported by under 50% of the respondents, they were as follows: low blood pressure, fever, chest pain, fainting, facial palsy (Bell’s palsy), dysphagia, and seizures. Of note, those with LD also reported experiencing hallucinations, mania, OCD, short-term memory problems, confusion, and tingling in the hands or feet.

In Figure 5, ~90 percent of survey respondents who reported a diagnosis of Rocky Mountain spotted fever experienced intermittent pain, anxiety, headache, brain fog, depression and short-term memory problems. All the other reported symptoms were below 90 percent. The following symptoms were reported with a frequency above 80 percent: headaches; arthritis, with severe joint pain and swelling; brain fog; extreme fatigue; depression; difficulty thinking, concentrating, and/or reading; GI/stomach problems; OCD; and panic/anxiety attacks. Of note, respondents also experienced hallucinations (68%), delusions (70%), neck stiffness, mania, and confusion. Over 40% of the respondents reported dysphagia, or difficulty swallowing. Similar to other TBDs, the following were reported by fewer respondents: facial palsy, fainting, and seizures.

PSS respondents reported a large variety of pain, neurological, and psychiatric symptoms. In addition to their symptoms, they also reported delayed and frustrating diagnostic experiences. Over 80% responded with negative sentiments when asked their opinion about their experiences with medical providers. When asked to detail their experiences in writing, the top negative sentiment score was associated with the following terms used by PSS respondents: doctors, believe, symptoms, listen, diagnose, and understand. These negative sentiments are associated with terms that equate to the burden of diagnosis and frustration with the medical community.

**Figure 6:**
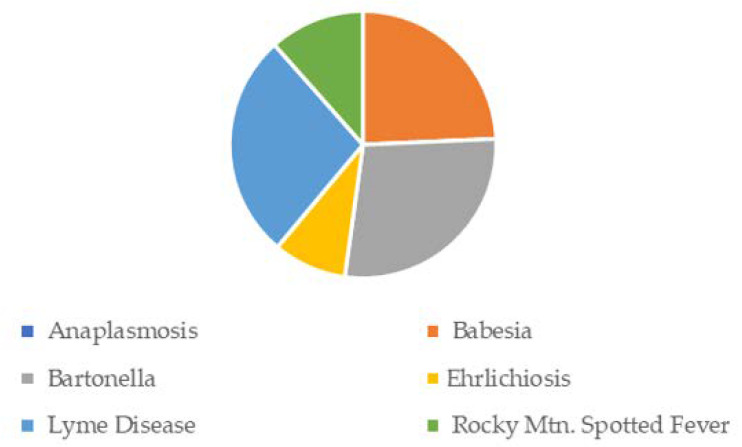
Facial/Bell’s Palsy Symptom Frequency, by TBD.

Given the number of available symptoms from which respondents could select, the most frequent were generally extreme fatigue, short-term memory issues, intermittent pain, headaches, depression, panic attacks, GI/stomach problems, and brain fog. Of note, more than half of the respondents reported unusual symptoms, such as hallucinations and delusions. Other frequently reported symptoms included nausea and vomiting, and neck stiffness. Surprisingly, approximately one-quarter of the respondents reported Bell’s palsy, which is often considered a classic sign of LD. Figure 6 details Bell’s palsy by TBD. Although bartonellosis was not included in the overall reporting, it is included below, given the large number of respondents who reported Bell’s palsy as a related symptom. Bartonellosis, LD, and babesiosis were the three most-reported TBDs, with Bell’s palsy at 21–26%. Bartonellosis was not included in the other charts or the results in this study due to the debate regarding tick transmission. The CDC does not include bartonellosis as a TBD in its official guidance to healthcare providers.

## 4. Discussion

### 4.1. Case Reports, Medical Literature, and Official Public Health Guidance

Through an extensive literature review, we explored the neuropsychiatric consequences of tick-borne diseases in the United States, using the symptoms and sequelae reported in the medical literature. The search revealed numerous neuropsychiatric case reports and documentation of symptoms not commonly reported by the Centers for Disease Control and Prevention in the reference manual for medical providers. Especially noteworthy is the disconnect between the scholarly literature regarding the psychiatric manifestations of LD and the lack of inclusion by the CDC of psychiatric symptoms, other than “altered mental status” in some TBD’s. The lack of concordance between the scholarly literature and the official guidance may add to the burden of diagnosis for patients presenting with psychiatric symptoms, specifically. These results suggest algorithms for specific TBDs could be developed for appropriate clinical diagnoses. Additionally, the clinical case definition needs to be expanded or broadened to reflect the growing understanding of TBDs, as reflected in the accumulating scientific, peer-reviewed, published literature.

Diagnostic delay for patients with a developing or lingering illness is costly and frustrating for patients and caregivers; burdens healthcare systems and resources when patients continue to seek a diagnosis; and incurs unnecessary costs for insurers. The present study surveyed TBD respondents to provide additional insight on the complex nature of diagnostic struggles, symptoms, and symptom severity found in the medical literature. In light of the number of neurological, pain, and psychiatric symptoms among patients with TBDs, which have been discovered in the scientific and case reports and which have limited overlapping symptom reports from official public health sources for healthcare providers, further study regarding neuropsychiatric symptoms and pain in TBDs is needed.

### 4.2. TBDs and Disease Progression, and Importance of Early Detection

Lyme and other TBD sequelae can present in all systems of the human body [138]. Early stages of Lyme disease occur between 1 and 30 days and are accompanied by viral-like illness, such as fatigue, fever, chills, myalgia (or joint and muscle pain), and headaches [139]. As the disease progresses to the early-disseminated period (1–4 months), symptoms worsen and arthritis (e.g., monoarticular and oligoarticular), fatigue, vision changes, and other problems develop for a patient. Late-stage disseminated Lyme disease can occur months and years after the initial tick bite and also after initial treatment and can include a host of debilitating multisystem symptoms [140]. Lyme and tick-borne disease patients may also develop encephalomyelitis or peripheral neuropathy [139]. Evidence from recent scholarship supports patient accounts of mental health challenges as a result of a TBD. Neuropsychiatric Lyme disease, caused by the pathogen Lyme borreliosis, presents as follows:

*significant comorbidity which may include developmental disorders, autism spectrum disorders, schizoaffective disorders, bipolar disorder, depression, anxiety disorders (panic disorder, social anxiety disorder, generalized anxiety disorder, posttraumatic stress disorder, intrusive symptoms), eating disorders, decreased libido, sleep disorders, addiction, opioid addiction, cognitive impairments, dementia, seizure disorders, suicide, violence, anhedonia, depersonalization, dissociative episodes, derealization and other impairments.*

[67]

Diagnostic burdens become a further challenge in late-disseminated LD and other TBDs. Maxwell [3] surveyed patients who had been diagnosed with LD and reported that not one was diagnosed by a psychiatrist, even though the vast majority reported psychiatric-related symptoms. Horowitz and Freeman noted the following: “Neuropsychiatric symptoms may result from and/or worsen when Lyme disease and associated coinfections, such as *Bartonella* spp. and *Babesiosis* spp., are present” [114].

As Lyme disease and other tick-borne diseases progress, physicians may overlook seemingly unrelated symptoms and sequalae, and may implicate purely psychiatric causes, rather than recognizing that neurologic and psychiatric symptoms are two prominent presenting characteristics of the progressing disease.

### 4.3. Patient Symptom Survey

The PSS supports the findings in the literature and medical case reports that suggest that neurological, psychiatric, and pain symptoms accompany all TBDs, but that are not acknowledged by official public health sources. Arthritic and neurological-related pain were prominent symptoms and included a wide-ranging list of unusual presentations. Difficulty swallowing was one neurological manifestation that was reported by a large number of patients who self-reported a TBD. Vertigo was also noted in significant numbers among the PSS respondents. However, pain and psychiatric symptoms were dominant presentations across all TBD diagnoses, with a focus on anxiety, depression, and panic attacks. Additional reported symptoms also included symptoms not acknowledged by public health, including delusions, hallucinations, and OCD. Bell’s palsy, often noted as a classic sign of LD, was reported less frequently by the respondents than anticipated, given the official CDC guidance.

## 5. Conclusions

Medical practitioners in the U.S. are not adequately alerted by official sources to the myriad—and often inconsistent—range of symptoms that may accompany many tick-transmissible illnesses. The literature and survey respondent data unite in offering an encompassing descriptive approach to TBDs that can augment public health knowledge, and the picture that it paints of patients with TBDs points to possible painful and poor quality of life health outcomes. The comprehensive literature review, combined with focused observational data, suggests that patients suffer at much higher rates than indicated in official public health reports.

Left untreated, TBDs can become chronic. Without a change in approach, this picture also conveys a bleak outlook for early and proper diagnosis. Negative patient sentiment related to the PSS respondents’ medical experiences demonstrates frustration with the burden of obtaining a diagnosis. Given inadequate testing, the multitude of non-specific patient symptoms, and the number and range of neuropsychiatric presentations that do not align with public health guidance, this study suggests the need for a revised approach to TBD diagnosis and improved communication from official public health sources regarding the broad symptomology associated with TBDs. Neuropsychiatric symptoms, depending on the context, should alert practitioners that there might be an infectious etiology.

The limitations of this study include possible inaccuracies regarding self-reported symptoms based on patient recall, especially among those who are ill; independent confirmation of patient self-reports were not included in the study. Survey respondents could have other conditions or illnesses that contribute to symptoms. This study was exploratory and designed to prompt further discussions regarding patient symptoms. Future research should include confirmation to rule out other illnesses or possible misrepresentation by the survey respondents. However, comparison of patient reports with an in-depth review of the neuropsychiatric TBD literature suggests that patient self-reports align with case reports and medical studies.

In summary, an expanded or broadened case surveillance definition, including neuropsychiatric and neurologic features—although they may not be present in every patient—would help to alert practitioners to the broader range of tick-borne disease manifestations.

## Figures and Tables

**Table 1:** Headache is a common feature of many TBDs. It often presents with other neurological symptoms. Case reports and scholarly literature indicate headache occurs in LD, Borrelia miyamotoi disease (BMD), and Powasan Virus, among others.

Occurs in early stages of LD. Common with other symptoms, such as fever, myalgia (muscle pain), arthralgia (joint stiffness), fatigue, flu-like symptoms, cranial nerve palsies, neck-stiffness, and meningitis. A headache is one of the most common manifestations of LD.	[6,12,36,37]
RMSF can cause a severe headache that is typically accompanied by a host of symptoms, including, nausea, vomiting, extreme fatigue, fever, and pain.	[7]
Meningitis, and associated headaches, is a neurologic expression of early-disseminated LD.	[7,38,39]
A headache is a clinical presentation in the co-infection, babesiosis, which is a parasite transmitted by the same tick that transmits LD. Babesiosis causes a malaria-like illness.	[40]
Headaches are common among patients infected with Bartonella species, specifically *B. henselae*. Headaches are reported with additional neurological symptoms, including memory loss, numbness, or a loss of sensation and balance problems.	[41,42]
Borrelia miyamotoi disease (BMD) symptoms include the following: fever, chills, headache, myalgia, arthralgia, malaise, and fatigue. Presentation of BMD is similar to human anaplasmosis and a differential diagnosis is warranted.	[12,43]
In human ehrlichiosis, headaches presents with fever, malaise, myalgia, and numerous multisystem presentations. Meningoencephalitis occurs in 20% of patients.	[44,45]
A headache is a clinical feature of tularemia.	[46]
Eighty-two percent of patients with anaplasmosis reported a headache.	[47]
A case report of a man in his sixties with a headache and non-focal weakness showed Powassan virus disease (PVD) in a serum analysis.	[48]
Headaches are reported by the CDC in the following TBDs: Lyme disease, babesiosis, Borrelia miyamotoi disease (severe headache), Heartland virus, anaplasmosis, Bourbon virus disease, Rocky Mountain spotted fever (severe headache), PVD, rickettsiosis, Tick-borne relapsing fever, tularemia, Colorado tick fever, Rickettsia parkeri rickettsiosis, and ehrlichiosis.	[27]

**Table 2:** Confusion/Altered Mental Status. Altered mental status is found in patients with ehrlichiosis, LD, TBRF, and Powassan, and anaplasmosis, among others. Numerous clinical reports indicated confusion and altered mental status as symptoms of TBDs.

Approximately 20% of patients with human monocytic ehrlichiosis (HME) present with neurological manifestations that include an altered mental status. Seizures may also be present.	[49]
Soldiers participating in summer exercises at Fort Chaffee, Arkansas, seroconverted to rickettsiae. Although some were asymptomatic, others presented with confusion, accompanied by dyspnea, myalgia, fever, and chills.	[44,50]
A case report found that an altered mental status was the sole manifestation of the central nervous system of early LD in an 84-year-old man.	[51]
Disorientation is a clinical manifestation of Lyme disease.	[52]
Confusion is a clinical feature of TBRF.	[46]
Confusion is a neurological symptom of anaplasmosis.	[7]
Confusion is noted in 20% of ehrlichiosis cases. “In a review of 21 patients,” (Ratnasamy, et al., 1996), “the most common clinical finding that predicted CSF abnormalities was a change in mental status. A total of 14 patients underwent computerized tomographic studies, and none of these studies showed abnormalities. Four (19%) of the 21 patients with CNS manifestations of ehrlichiosis and abnormal CSF findings died”.	[7,53,54]
An 87-year-old man, presenting with an altered mental status, tested positive for three tick-borne diseases, specifically, Powassan virus encephalitis, severe babesiosis, and Lyme disease (Lyme carditis).	[55]
A 74-year-old woman with an affinity for gardening “presented with one week of progressive dyspnea, cough with mucoid expectoration, and fatigue. On presentation, she was afebrile, hypotensive, and tachycardic. General examination was significant for altered mental status, dyspnea, pallor, and peripheral edema”. A peripheral blood smear revealed babesiosis. She also had titers for anaplasmosis.	[56]
The CDC lists confusion as an uncommon symptom of Borrelia miyamotoi disease. Altered mental status is a symptom of ehrlichiosis from all the following agents: Ehrlichia chaffeensis, Ehrlichia ewingii, and Ehrlichia muris eauclairensis; Powassan virus disease (PVD); RMSF (after five days); Tick-borne encephalitis (TBE); and babesiosis from both agents, babesiosis microti, and other babesiosis species.	[27]

**Table 3:** Pain may present as rheumatological, muscle-skeletal, or neurological. The present study asked survey respondents about pain severity in general. Below, pain is covered with a focus on neurological and also in general, including arthritis, joint, and muscle pain, which tend to present as common symptoms with other frequently occurring symptoms, such as headache and fatigue.

“Neurogenic pain with radiculitis is often the starting symptom in adult patients with tick-borne Lyme neuroborreliosis and in some cases the only clinical manifestation”.	[57]
In all of the 41 patients with neuroborreliosis that were examined, pain presented early and was a prominent symptom.	[58]
In a systematic review, the authors found that arthralgias (joint pain) is “the most common form of pain associated with Lyme arthritis”. Acute nervous system involvement also involves acute pain in Lyme disease. The pain is typically neuropathic, aching, radicular, and can be worse at night.	[57,59]
Lyme arthritis may produce knee pain in up to 60% of cases of Lyme borreliosis in North America.	[59,60]
One to three weeks after a tick bite in human granulocytic anaplasmosis (HGA), patients present with fever, chills, “faintness, or generalized musculoskeletal pain with headaches and myalgia”.	[61]
In a systemic review, and based on 21 studies, exposed patients with LD were more likely to have the following: neck pain, myalgia, and arthralgia. These symptoms overlapped with paresthesia, sleep disorder, poor appetite, and concentration difficulties. Neck pain, myalgia, and arthralgia were the three most common overlapping symptoms among exposed patients in North America and Europe.	[62]
In a case report of a 16-year-old boy in Missouri who presented with fever and myalgia, he was diagnosed with ehrlichiosis.	[63]
Myalgias is commonly reported in cases of tularemia, following an incubation period of 3–5 days, and is accompanied by fever, malaise, and headaches, among other symptoms.	[7]
Lyme radiculoneuritis presents with severe and deep muscle pain, which is often asymmetric and worse at night.	[7]
Myalgia is common in almost every tick-borne disease, including babesiosis, RMSF, tularemia, and ehrlichiosis, according to the *American Family Physician*.	[64]
Myalgia is a clinical feature associated with the following: Anaplasma phagocytophilum, Ehrlichia chaffeensis, Ehrlichia ewingii, Lyme disease (*B. burgdorferi*), *B. miyamotoi*, Powassan virus, and Heartland virus.	[16]
The CDC reports the following diseases associated with pain: Babesiosis–abdominal pain; Borrelia miyamotoi disease–abdominal pain; ehrlichiosis–muscle pain; Lyme disease–migratory pain in tendons, bursae, muscle, and bones; tularemia–abdominal pain, severe throat pain, and pleuritic chest pain.	[27]

**Table 4:** Scholarship related to LD or tick-borne disease and seizures appears in individual case reports more frequently than population studies. Official recognition by the CDC of seizures in tick-borne disease are noted only in Powassan virus disease and tick-borne encephalitis virus (TBE).

Progressive inflammatory reactions are related to seizure activity. In LD, a psychoimmune process is associated with psychiatric symptoms in LD patients. “Borrelia burgdorferi infections have been associated with the proinflammatory cytokines IL-6, IL-8, IL-12, IL-18 and interferon g, the chemokines CXCL12 and CXCL13 and increased levels proinflammatory lipoproteins”.	[65]
There was a case report of a patient presenting with Lyme cerebral vasculitis and grand mal seizures. Stroke and stroke-like syndromes can also be associated with LD.	[66]
Comorbidities result from immune and metabolic effects as LD progresses, leading to the gradual development of a host of neuropsychiatric symptoms. These can include seizures, depersonalization, suicide, anxiety disorders, eating disorders, depression, autism spectrum disorders, and other neuropsychiatric sequelae.	[67]
A previously healthy 13-year-old boy presented with seizures. Through serological testing, the patient was diagnosed with neuroborreliosis and improved with three weeks of ceftriaxone.	[68]
In death certificates with LD as the underlying cause of death, there was one report of a seizure disorder being the terminal event.	[69]
A case report of an 18-year-old male presenting with seizures and a headache had a positive serum analysis for PVD.	[48]
The Centers for Disease Control and Prevention recognize seizures as a symptom in PVD and tick-borne encephalitis virus.	[27]

**Table 5:** Vertigo and/or dizziness may not be fully recognized as a TBD symptom. Medical literature and case reports demonstrate that vertigo is present in cases of LD and TBRF.

Can present as the sole symptom in later stage or chronic LD.	[70]
In a study of 38 patients with confirmed LD, vertigo was found to be a symptom of LD. LD can result in labyrinthitis and hearing-organ damage.	[71]
A study of 266 LD patients presented most commonly with headaches, a stiff neck, and dizziness.	[72]
In a study of 73 patients, “Borrelia infection is an etiological factor which should be considered in patients suffering from vertigo especially if positional nystagmus is present”.	[73]
As LD progresses to the second stage, the primary symptoms become malaise, fever and chills, headache, myalgias, arthralgias, and dizziness.	[74]
Dizziness, often accompanied by confusion, is a clinical manifestation of TBRF.	[46]
According to the CDC, vertigo is uncommon and only present with Borrelia miyamotoi.	[27]

**Table 6:** Tingling and numbness tend to occur in the extremities and are associated with LD in case reports.

A 72-year-old man, with a history of a bite two weeks earlier, presented with numbness and tingling. He tested positive for Borrelia burgdorferi, via a western blot and enzyme-linked immunosorbent assay. The patient was diagnosed with transverse myelitis from Lyme disease and babesiosis.	[75]
As LD progresses, patients can present with a combination of neurological symptoms. These can include the following: memory loss; inability to concentrate; and muscle weakness, with tingling and numbness in the arms and legs.	[76]
Tingling, numbness, and pain, particularly on the extremities, are common features of LD, particularly in the second and third stages.	[77,78]
A case report of a 31-year-old man with a dual diagnosis of Lyme and Guillain–Barre syndrome (GBS), presented with progressive numbness in his hands and feet. The symptoms resolved with intravenous immunoglobulin and parenteral ceftriaxone.	[79]
The Centers for Disease Control and Prevention does not list tingling or numbness as symptoms of LD or any TBD in the annual report.	[27]

**Table 7:** Cognitive function (concentration, word finding, and memory difficulty)–limited literature is available regarding cognitive function among TBD patients in the United States. Concentration tends to be related to Tick-borne encephalitis, which is endemic in Europe and Asia. The following studies are reported from the US.

An inability to concentrate develops with Lyme disease’s progression and is accompanied by other neurological symptoms.	[76]
Short-term memory deficits progress with LD and are accompanied by other neurological symptoms, such as tingling and numbness.	[76]
In a systematic review of exposed patients in North America and Europe, the symptoms of those with North American strains of Lyme disease were more cognitive-related, with 10–20% of these patients experiencing cognitive symptoms and fatigue more than six months after antibiotic treatment.	[62]
“Up to 46% of patients [with TBD] are left with permanent sequelae at long-time follow-up, the most commonly reported residuals being various cognitive or neuropsychiatric complaints, balance disorders, headache, dysphasia, hearing defects, and spinal paralysis”.	[80]
The Centers for Disease Control does not list concentration or memory as symptoms of LD or any TBD.	[27]

**Table 8:** Paralysis: difficulty swallowing (dysphagia) and Bell’s palsy. This exploratory study was designed to capture different sources of information and assess the possible disconnect among them, with respect to patient symptoms. Dysphagia is one symptom with which medical providers may not be familiar, but self-reports or case reports in the medical literature might suggest that the symptom may require further exploration. Unlike dysphagia, Bell’s palsy is recognized by official public health sources and is suggested as a recognized symptom of LD in approximately eight percent of cases [81].

In a case report of an 87-year-old hunter with Lyme neuroborreliosis (LNB), dysphagia occurred as a consequence of facial weakness. The patient’s dysphagia and other symptoms improved with intravenous ceftriaxone after two weeks. The patient was readmitted with a cough and dyspnea. This case suggests that acute respiratory failure can develop from diaphragmatic paralysis.	[33]
North American tick paralysis (TP) can present with cranial nerve, which may include drooling, dysphagia, and facial weakness.	[82]
Neuroborreliosis includes multiple neurological symptoms, including dysphagia. In a case report of a 76-year-old woman with LB, the woman received two weeks of IV Ceftriaxone. Her confusion improved rapidly, while her dysphagia gradually improved.	[83]
Five hundred and fifty-nine patients presented with facial palsy in a Netherlands hospital-based study and 4.7% (26) of them had LNB. Of these, over 70% of LBN patients with facial palsy did not report a tick bite or rash. LNB patients with facial palsy were more likely to occur in the summer months. LBN patients with facial palsy presented more often with headaches, than non-LBN-palsy patients.	[84]
Bell’s palsy can be bilateral and is the most common LD neurological symptom in children. Intermittent and continuing arthritis, and, less often, encephalopathy or neuropathy are also common.	[16]
Kim et al. (2012), reported a case of a “32-year-old man with rapidly progressive bilateral ptosis, dysphagia, spastic paraparesis, and voiding difficulty in whom Lyme disease was diagnosed through serologic tests for antibodies and Western blot testing”.	[85]
In LD, the peripheral nervous system is typically affected more than the CNS, this includes Bell’s palsy.	[7]
A patient presented with Bell’s palsy, in addition to numerous other symptoms, including altered mental status and multisystem organ involvement. The patient was found to have ehrlichiosis.	[86]
The CDC reports facial palsy in the following: TBRF, but notes it is very rare; and Bell’s palsy and other cranial neuropathy are noted as LD symptoms. The CDC does not report dysphagia as a symptom of any TBD.	[27]

**Table 9:** Difficulty with, or slurred speech (dysarthria). Dysarthria is a neurological manifestation of a number of tick-borne diseases. Numerous case reports link difficulty with speech or slurred speech to anaplasmosis, LD, and PVD. The CDC does not report dysarthria as a symptom of any TBD, indicating more research is needed with this neurological manifestation.

A 63-year-old woman presented with the following: dysarthria (slurred speech), fever, confusion, and new-onset thrombocytopenia, two weeks after a tick bite. She was diagnosed with human granulocytic anaplasmosis (HGA).	[87]
A 63-year-old male presented with fever, dysarthria, and dysphagia after receiving multiple tick bites on a recent vacation. Symptoms were accompanied by the following: lymphopenia and thrombocytopenia. Laboratory findings concluded that the patient had human granulocytic anaplasmosis (HGA).	[88]
Dysarthria occurs in acute illness, among short- and long-term survivors, and among hospitalized patients with Powassan virus disease (PVD). The incubation period for PVD is 1–4 weeks.	[89,90]
Sequelae in PVD survivors occurs with mutism, and in the long-term, with anarthria (a severe form of dysarthria) and aphasia (loss of ability to understand or express speech).	[91,92,93]
Apnea and psychoses present with dysarthria, as short-term neurological sequelae with PVD. Seventy-five percent of PVD cases include significant neurological complications.	[89]
Dysarthria was the only consequence of neuroborreliosis in a case report of a 65-year-old man.	[94]
Case reports of dysarthria are found in rickettsial infections of granulocytes. Human granulocytic anaplasmosis (HGA) is caused by the intracellular bacterium, Anaplasma phagocytophilum.	[88,95]
A 46-year-old man was admitted to hospital with dizziness, fever, and trouble walking. The symptoms developed four months after visiting an endemic area. The patient was diagnosed with Lyme neuroborreliosis and, when examined, had dysarthria, ataxia (uncoordinated movements), and hemianopsia (blindness in half of his vision).	[96]
In a study of 30 LD patients with LD or “Lyme-like” disease in Brazil, dysarthria occurred in 6.7%.	[97]
A 66-year-old woman presented to the hospital with slurred speech, lethargy, and ataxia (loss of coordination). She was confirmed to have Ehrlichia chaffeensis.	[49]
The Mayo Clinic lists dysarthria as a symptom of LD, but not in relation to other TBDs. The National Organization for Rare Diseases (NORD) reports dysarthria in Rocky Mountain spotted fever (RMSF).	[98,99]
The CDC does not report difficulty with speech or slurred speech, or dysarthria as symptoms associated with Lyme disease or other TBDs.	[99]

**Table 10:** Low blood pressure (hypotension). Hypotension is associated with autonomic nervous system dysfunction and is demonstrated primarily with ehrlichiosis in the case reports. The National Organization for Rare Diseases (NORD) recognizes hypotension with ehrlichiosis and RMSF, but the CDC does not link this symptom to any TBD.

A patient presented with life-threatening hypotension from babesiosis hemolysis.	[100]
Chronic babesiosis includes life-altering symptoms similar to malaria, including hypotension and headaches.	[101]
Profound hypotension is associated with human monocytotropic ehrlichiosis (HME), caused by the bacterium *Ehrlichia chaffeensis*. It is also associated with human granulocytotropic anaplasmosis (HGA), caused by *A. phagocytophilum*.	[102,103]
There was a case study of a 52-year-old man who had been hiking in Georgia and was bitten by a tick, who then presented to the ER with a fever and low blood pressure. He tested positive for HME. A similar case occurred with a man from Tennessee, who presented with a fever and low blood pressure. He also tested positive for HME.	[104]
TBRF can lead to hypotension.	[46]
Low blood pressure (hypotension) is reported with human monocytic ehrlichiosis (HME) and RMSF by NORD.	[99]
Low blood pressure is reported as a complication of babesiosis by the CDC.	[27]

**Table 11:** Fainting (syncope). Syncope has been reported with Lyme disease and other tick-borne diseases. Although syncope, generally, may be associated with neurological conditions, it may have cardiovascular etiologies. In the TBD case reports, fainting tended to be associated with Lyme carditis, rather than neurological origins.

There was a case report in which a 55-year-old man was admitted to the hospital with flu-like symptoms, shortness of breath, near syncope, and bradycardia of 20–30 beats per minute. He had exposure to tick bites and tested positive for LD. He was diagnosed with Lyme carditis. “Considering Lyme carditis as a differential diagnosis in patients with an AV block of an unknown etiology can result in a timely diagnosis and treatment of Lyme carditis”.	[105]
“Clinical manifestations include syncope, light-headedness, fainting, shortness of breath, palpitations, and/or chest pain. Atrioventricular (AV) electrical block of varying severity presents the most common conduction disorder in Lyme carditis”.	[106]
There was a case report of a 20-year-old male who presented to an outpatient physician’s office after a five-minute episode of syncope. The patient had a rash and was an avid outdoorsman. He was found to have persistent bradycardia, a complete heart block, and Lyme carditis. Lyme disease was confirmed via two-tier testing.	[107]
A 53-year-old man from Delaware suffered from recurrent syncope and fevers, and thrombocytopenia and macroscopic hematuria. He was diagnosed and treated for Babesiosis with antibiotics and atovaquone, with his symptoms resolving within 48 h.	[108]
Five women, aged 22–44, had a history of LD, but were successfully treated with antibiotics. They later “developed symptoms of fatigue, cognitive dysfunction, orthostatic palpitations and either near syncope or frank syncope”. All were diagnosed with postural orthostatic tachycardia syndrome (POTS).	[109]
Three patients with Lyme borreliosis, who were previously healthy, developed syncope abruptly. All three also had an atrioventricular (AV) heart block.	[110]
Syncope, chest pain, and dyspnea occur in LD patients within 2–4 weeks of infection, but symptoms can take up to seven months to appear.	[16]
Syncope is not reported by the CDC as a symptom of any TBD.	[27]

**Table 12:** Depression is found in many patients with LD and is noted by a state-level public health report as a clinical feature of babesiosis.

In a study of post-treatment LD syndrome patients (PTLD) compared to health controls, those with PTLD were significantly more likely to have depression, using the Beck Depression Inventory-II scale.	[113]
Seventy-seven percent of LD patients reported having depression.	[114]
Depression is the most common symptom in LD patients, with psychiatric sequelae ranging from 22% to 66%.	[115,116]
The Michigan and New York State governments list depression as a clinical feature of babesiosis in their reference literature on emerging infectious diseases for healthcare providers.	[117]
In a study of patients with late-stage Lyme borrelia, “a significantly higher percentage of depressive disorders was...noted in the group of males and females with neuroborreliosis [in comparison to those with Lyme arthritis]”.	[118]
In a study of the self-reported symptoms of those diagnosed with LD, the most frequently noted symptoms were as follows: depression, extreme fatigue, headaches, neck and back pain, brain fog, and anxiety.	[3]
The CDC lists depression as uncommon, but possible in babesiosis. The CDC does not include depression as a symptom linked to any other TBD.	[27]

**Table 13:** Anxiety is not well studied in the medical literature as a symptom of TBDs, but is shown to be present with LD. TBRF is also associated with anxiety.

Sixty-seven percent of LD patients report having anxiety.	[114]
Neurological psychiatric symptoms develop gradually in patients with Lyme borreliosis (LB) and include anxiety disorders, such as panic disorders, social anxiety, general anxiety, and other anxiety disorders.	[67,119]
Anxiety is a clinical feature of bartonella, which is often recognized as a co-infection of Lyme disease.	[120]
TBRF can produce psychiatric symptoms, including anxiety, and patients should be monitored for anxiety during treatment.	[46]
“A higher level of risk to self and others is associated with multiple symptoms developing after acquiring [Lyme and Associated Diseases] LAD, in particular, explosive anger, intrusive images, sudden mood swings, paranoia, dissociative episodes, hallucinations, disinhibition, panic disorder, rapid cycling bipolar, depersonalization, social anxiety disorder, substance abuse, hypervigilance, generalized anxiety disorder, genital-urinary symptoms, chronic pain, anhedonia, depression, low frustration tolerance, and posttraumatic stress disorder”.	[121]
The CDC does not recognize anxiety as a symptom of any tick-borne disease.	[27]

**Table 14:** Fatigue and malaise. Fatigue (i.e., extreme tiredness) or malaise (i.e., lack of wellbeing) are often reported in the literature as primarily clinical features of Lyme and other tick-borne diseases, particularly in cases of human monocytic ehrlichiosis (HME). Although ample evidence supports fatigue and malaise as symptoms of LD, scholarly literature is burdened with debates regarding chronic LD or post-treatment Lyme disease syndrome (PTLDS). The findings, below, focus on the earlier stages of LD and other tick-borne diseases.

Fatigue is a clinical presentation of Lyme disease, Bourbon virus (BRB), and Heartland virus.	[16]
The majority of Lyme disease patients in the United States develop fatigue, which typically presents with headaches, an EM rash, a mildly stiff neck, and arthralgia or myalgia.	[122]
In a study of Pennsylvania’s health records, Lyme disease was found to be more prevalent than surveillance data, with “20.8% (n = 735) of cases with a diagnosis code and treatment had a diagnosis of malaise or fatigue, pain, or cognitive difficulties not present in the past 26 weeks”, specifically within 4–52 weeks following their Lyme disease diagnosis. There was “no prior evidence of chronic fatigue, fibromyalgia, or chronic pain based on diagnosis codes linked to encounters, medication orders, or problem lists”.	[123]
A 55-year-old man presented with AV block, fatigue, malaise, and flu-like symptoms. The case report concluded that “Lyme carditis [should be considered] as a differential diagnosis in patients with an AV block”.	[105]
An EM rash is often accompanied by the following clinical features: fever, malaise, headache, myalgia or arthralgia, regional lymphadenopathy, and a stiff neck.	[124]
In a prospective, double-blind study of 1156 males who had positive Borrelia antibodies, the authors found the following: “Seropositive subjects who had never suffered from clinically manifest Lyme borreliosis or neuroborreliosis showed significantly more often chronic fatigue (*p* = 0.02) and malaise (*p* = 0.01) than seronegative recruits”. They concluded as follows: “It is worth examining whether an antibiotic therapy should be considered in patients with chronic fatigue syndrome and positive Borrelia serology”.	[125]
Malaise is a common clinical presentation, typically with headaches, in ehrlichiosis.	[126]
In a population study of confirmed human granulocytic ehrlichiosis (HGE) cases, malaise, with fever, headaches, and myalgia, were the most common symptoms.	[127]
“The hallmarks of symptomatic human monocytic ehrlichiosis (HME) include fever, headache, myalgia, nausea, malaise, transaminitis, and blood cell abnormalities”. Two cases presented with the additional symptom of sudden sensorineural hearing loss.	[128]
Fatigue is a classic presentation in anaplasmosis, with other non-specific influenza-like symptoms, such as fever, fatigue, muscle aches, and headaches.	[129,130]
Babesiosis presents with typical clinical symptoms that include the following: fever, fatigue, malaise, weakness, chills, sweats, and headaches.	[131]
The CDC reports fatigue as present in the following TBDs: Borrelia miyamotoi, babesiosis, Colorado tick fever, Heartland virus disease, and tularemia. Malaise is reported in the following: RMSF, anaplasmosis, tularemia, babesiosis, ehrlichiosis, and Lyme disease, with malaise as “flu-like symptoms” accompanied by headaches, fever, myalgia, and arthralgia.	[27]

**Table 15:** Mania, panic attacks, delusions, or hallucinations. Tick-borne illnesses are noted by patients and advocacy organizations to cause psychiatric symptoms similar to bi-polar and schizophrenia, reportedly leading to unnecessary institutionalization and/or misdiagnosis. Official sources of information are lacking on these psychiatric manifestations, which may delay diagnosis, as medical providers are not informed via the public health system. Mania, panic attacks, hallucinations, and delusions have been combined in this section to cover the overlapping presence of the three clinical manifestations in the scholarly literature.

In a case report of a previously healthy 35-year-old woman, with no prior history of psychiatric disorders and no family history of mania, months of psychiatric symptoms developed after she refused continued treatment for LD. The woman had a presumed EM rash and lived in a Lyme-endemic area. Additional symptoms included severe fatigue, headaches, and an irritable mood. Her mania overlapped with auditory hallucinations and paranoid delusions. Episodes continued until she ultimately agreed to treatment with IV antibiotics, which resulted in an improvement in all the symptoms.	[132]
A 64-year-old woman was admitted to a psychiatric hospital with visual hallucinations. She also developed neurological symptoms and had an abnormal EEG. A lumbar puncture showed LB. She had a history of tick bites, and her husband had experienced similar symptoms. Medications for psychosis failed to help her improve.	[133]
A seven-year-old boy presented with Alice in Wonderland syndrome, with distorted perceptions of shapes and sizes, and he also had auditory hallucinations. His Lyme serology was positive in both the serum and cerebrospinal fluid. His symptoms dissipated after three weeks of intravenous ceftriaxone.	[134]
Musical hallucinations occurred in two female patients with neurologic Lyme disease. The case reports suggest the following: “Patients with musical hallucinations of unknown cause should be tested for infection with the Lyme disease spirochete”.	[135]
A patient presented with “no neurological signs but marked psychiatric symptoms induced by borrelia burgdorferi, whose clinical picture was indistinguishable from an endogenous schizophrenia…The case demonstrated the aetiologic nonspecificity of paranoid symptoms and hallucinations and emphasizes that in psychotic patients without psychiatric history additional diagnostic measures should be performed”.	[136]
Three patients with panic-like episodes were also described and were found to have tick-borne diseases. “Each woman experienced symptoms that are not usual in panic disorder but are typical of neurological Lyme disease, including exquisite sensitivity to light, touch, and sounds, joint pain often in combination with cognitive changes including mental fogginess and loss of recent memory, and some degree of bizarre, shifting, and often excruciating neurological pain”.	[137]
The CDC does not report mania, panic attacks, delusions, or hallucinations as symptoms or sequalae of any TBD.	[27]

**Table 16:** CDC Tickborne Diseases of the United States: A Reference Manual for Healthcare Providers (2018). Summary of all TBD Symptoms.

Symptom	LD	Ehrlichiosis	Babesiosis	Anaplasmosis	CTF	RMSF	BMD	HVD	PVD	R Parkeri	TBRF	Tularemia
Rash	•	in children		uncommon	•	•	uncommon			•		
Fever/Chills	•	•	•	•	•	high	•	•	•	•	•	•
Fatigue	•	•	•	•	•		•	•	•			•
Malaise	•	•	•	•		•						•
Headache	•	•	•	severe	•	severe	severe	•	•	•	•	•
Myalgia	•	•	•	•	•	•	•	•		•	•	•
Nausea/Vomiting		•	•	•		•	uncommon	•	•		•	•
Shakes/Rigors				•								
Diarrhea		•		•			uncommon	•				•
Anorexia	•	•	•		•	uncommon					•	
Arthralgia	•		•				•	•			•	•
Dark urine			•				•					
Confusion							•					
Vertigo/Dizziness							uncommon					
Dyspnea							uncommon					
Pharyngeal erythema	•				•							
Lymphadenopathy	•				•							•
Altered mental status		•	•		•	•			•			
Decreased appetite								••				
Baker’s cyst	•											
Conduction abnormalities	•											
Myocarditis/Pericarditis	•											
Bell’s palsy or other cranial neuropathy	•	•	•		•				•		•	
Meningitis	•								•			
Motor and sensory radiculoneuropathy, and mononeuritis multiplex	•											
Subtle cognitive difficulties	•											
Encephalitis, encephalomyelitis, subtle	•								•			
encephalopathy, and pseudotumor cerebri (all rare)	•											
Paresis									•			
Seizures									•			
Aphasia (loss of speech)												
Multi-organ system damage						•						

CTF—Colorado tick fever; RMSF—Rocky Mountain spotted fever; BMD—Borrelia miyamotoi diseases; HVD—Heartland virus disease; PVD—Powassan virus disease; TBRF—Tick-borne relapsing fever.

**Table 17:** Symptom comparison from medical literature and the public health reference manual for common TBDs.

Symptom	Reported in Scientific and Medical Literature	Reported by the Centers for Disease Control	Reported in the Scientific Literature, but Not Recognized by the CDC
Headache	LD, BVD, babesiosis, ehrlichiosis, tularemia, anaplasmosis, RMSF, and PVD	LD, ehrlichiosis, babesiosis, anaplasmosis, RMSF BMD, PVD, and tularemia	None, headache is the most common presenting neurological symptom among all TBDs
Confusion/Altered Mental Status	LD, babesiosis, ehrlichiosis, anaplasmosis, and PVD	Confusion: BMD Altered mental status: ehrlichiosis, babesiosis, RMSF, and PVD	LD anaplasmosis
Pain	LD, babesiosis, ehrlichiosis, anaplasmosis, tularemia, RMSF, and PVD	LD, Babesiosis, ehrlichiosis, anaplasmosis, tularemia, RMSF, and PVD	PVD
Seizures	LD, RMSF	PVD	LD RMSF
Vertigo/Dizziness	LD, RMSF	None	LD RMSF
Tingling/Numbness	LD	None	LD
Cognitive Function (concentration, memory difficulty, and word recall)	LD	None	LD
Paralysis: difficulty swallowing (dysphagia) or Bell’s palsy	Bell’s palsy: LD, ehrlichiosis, and dysphagia: LD	Bell’s Palsy: LD, ehrlichiosis, babesiosis, and dysphagia: none	Dysphagia: LD
Difficulty with, or slurred speech (Dysarthria)	LD, anaplasmosis, ehrlichiosis, PVD, and RMSF	None	LD, anaplasmosis, ehrlichiosis, PVD, RMSF
Low Blood Pressure (hypotension)	babesiosis, ehrlichiosis, and anaplasmosis (also present in TBRF)	Babesiosis	Ehrlichiosis, anaplasmosis, and TBRF
Fainting (syncope)	LD, babesiosis	None	LD, babesiosis
Depression	LD, babesiosis	Babesiosis, but uncommon	LD
Anxiety	LD (also present in TBRF)	None	LD
Fatigue and malaise	LD, ehrlichiosis, anaplasmosis, and babesiosis	LD, Ehrlichiosis, anaplasmosis, babesiosis, and tularemia (and other TBDs)	None, fatigue and malaise are commonly agreed upon as classic symptoms of TBDs
Mania, panic attacks, delusions, or hallucinations	LD	None	LD

**Table 18:** Respondent Characteristics by Diagnostic Category. Summary statistics for the number of respondents, and their age and sex, who reported a tick-borne disease are provided for each disease (i.e., by diagnostic category).

Diagnostic Cohort	Has Diagnosis	Age		Female
Statistics Presented	n (% of Analytical Cohort)	Mean (Standard Deviation)	Median (Inter-Quartile Range) [Minimum–Maximum]	n (% of Diagnostic Cohort) (% of Analytical Cohort)
Anaplasmosis	7 (4.73%)	48.14 (13.85)	44 (31) [34–68]	5 (71.43%) [3.38%]
Babesiosis	22 (14.86%)	40.09 (16.27)	44 (20) [9–67]	17 (77.27%) [11.49%]
Bartonella	23 (15.54%)	42.17 (14.91)	44 (22) [9–67]	20 (86.96%) [13.51%]
Ehrlichiosis	12 (8.11%)	43.92 (18.70)	48.5 (21.5) [9–69]	9 (75%) [6.08%]
Lyme Disease	43 (29.05%)	45.58 (15.87)	45 (25) [9–79]	39 (90.7%) [26.35%]
Rocky Mountain Spotted Fever	37 (25.00%)	51.46 (12.84)	54 (21) [25–73]	31 (83.78%) [20.95%]

## Data Availability

Data is available upon request.
